# Efficacy of Hydrogen Purification and Cosmetic Acids in the Treatment of Acne Vulgaris: A Preliminary Report

**DOI:** 10.3390/jcm11216269

**Published:** 2022-10-25

**Authors:** Karolina Chilicka, Monika Rusztowicz, Aleksandra M. Rogowska, Renata Szyguła, Binnaz Asanova, Danuta Nowicka

**Affiliations:** 1 Department of Health Sciences, Institute of Health Sciences, University of Opole, 45-040 Opole, Poland; 2 Department of Social Sciences, Institute of Psychology, University of Opole, 45-052 Opole, Poland; 3 Medical College Yordanka Filaretova, Medical University of Sofia, 1606 Sofia, Bulgaria; 4 Department of Dermatology, Venereology and Allergology, Wrocław Medical University, 50-368 Wrocław, Poland

**Keywords:** acne vulgaris, hydrogen purification, chemical peels, Sebumeter, Corneometer, cosmetology

## Abstract

Acne and skin lesions that appear in its course deteriorate the quality of life of patients, cause depression and the emergence of suicidal thoughts. Cosmetic treatments can have a positive effect on improving skin condition by cleaning up skin eruptions, thus improving the well-being of affected people. Hydrogen purification is a treatment that uses alkaline water generated by a device, which reduces sebum from the surface of the epidermis. This is a novel treatment that has recently been introduced to beauty salons. On the other hand, cosmetic acids have been used for many years for treating people with acne vulgaris and give spectacular results in terms of improving the skin condition. In this study, skin condition was evaluated with a Derma Unit SSC 3 device. The Global Acne Grading System (GAGS) was used to check acne severity. Twenty-four women aged 19–21 years (*M* = 20.13, *SD* = 0.80) diagnosed with mild acne vulgaris and a high sebum level participated in the study. Group A underwent a hydrogen purification treatment using an H2jet manipulator, which ejected alkaline water from the manipulator under pressure. Group B underwent a hydrogen purification treatment with the use of a phytic, pyruvic, lactic and ferulic acids at 40% mixture (pH 1.4). A series of four treatments was performed at 14-day intervals in both groups. Skin parameters were measured before and 30 days after the series of treatment. Very good results were obtained in both groups. The skin eruptions in patients were reduced and we also observed lower amounts of sebum on the surface of the epidermis, and an improvement in skin hydration. However, in group B, the results were better than in group A. The study showed that the synergy of the treatments produced much better effects than those obtained by completing the hydrogen purification treatment alone.

## 1. Introduction

Acne vulgaris is a chronic disease. Several types of lesions can occur during acne, including non-inflammatory and inflammatory lesions, scars, rush, and discolorations. Comedones are classified as non-inflammatory lesions. They can be microscopic (micro-blackheads) and serve as precursors of open or closed blackheads visible on the skin. The causes of acne revolve around the interplay of several factors, including increased sebum production, follicular hyperkeratosis, inflammation, and the action of anaerobic *Cutibacterium acnes* in the hair follicles [1,2,3,4].

Acne vulgaris is a common skin disease that affects teenagers and young adults. It is estimated that 80% to 90% of adolescents experience varying degrees of acne symptoms, which may continue into adulthood. Exacerbation of acne may depend on premenstrual flares, diet, and body mass index (BMI) [5,6]. Long-term treatment of acne, the constant appearance of skin eruptions, and acne scars can negatively affect mental and physical health. The presence of this disease causes discomfort that can lead to emotional disorders, reduced quality of life, and depression [7,8,9,10]. However, despite many years of research, the pathogenesis of acne vulgaris has not yet been fully elucidated, and effective treatments have not yet been developed.

Hydrogen is formed by two hydrogen atoms, and it is an odorless and colorless gas that dissolves in water. It builds most of the organic molecules and takes third place in terms of the number of elements found in the human body. Hydrogen is 1000 times smaller than bacteria and cells in the human body with a molecule size of 0.24 nm. Furthermore, it diffuses quickly, so it easily penetrates the structures of the human body and the skin barrier.

Alkaline water has recently begun to be used in cosmetology. In the hydrogen purification treatment, alkaline water, namely electrochemically reduced water (ERW), is used. Such water is rare in nature, but it can be produced by an instant chemical process called electrolysis; the pH of such water is 8–10. During the electrolysis process, by which a direct current is passed between two electrodes (anode and cathode) separated by a semi-permeable membrane, the elements contained in the water are broken down into hydrogen ions (H^+^) that are gathered around the cathode and hydroxyl ions (OH^−^) that are gathered around the anode. H^+^ ions form ionized alkaline water. Negative OH^−^ ions form acidic water. Active molecular hydrogen that is present in alkaline water serves as the main factor in building the ORP. Its level is related to the concentration of alkaline water and falls within the range of 0.3–0.6 mg/L. Water with hydrogen ions is a natural antioxidant and can be produced solely during electrolysis (water ionizers) and hydrogen saturation (hydrogen generators). Recently, hydrogen cleansing has become one of the most popular treatments in cosmetology, which is used to reduce free radicals, and thus has an anti-aging effect. Once introduced into cells, the hydrogen atoms donate their electrons to free radicals, which are converted into water molecules. In this way, the activity of free radicals is neutralized [11].

In 2007, Ohsawa et al. [12] demonstrated that molecular hydrogen can selectively reduce reactive forms of oxygen in vitro and exert antioxidant, anti-inflammatory, and anti-apoptotic effects. For many years, scientists have been researching the internal effects of alkaline water in people with problems such as pyrosis, dysphoria, tympanites, and diarrhea. The research results confirm that drinking alkaline water significantly improves health conditions [13,14,15,16].

Cosmetic acids have been the basis for proper care of acne-prone skin, among others, for years. They cause exfoliation of the dead epidermis, thanks to which the skin pores are cleansed, the incidence of inflammatory skin eruptions is reduced, and the secreted sebum is reduced on the surface of the epidermis. Cosmetic acid treatments can complement dermatological therapies. The acids most commonly used for acne skin include ferulic, azelaic, mandelic, glycolic, salicylic, pyruvic, lactic, and phytic acids. However, combining different topical therapies may produce more beneficial results in treating problematic skin. An extremely important point is also the close cooperation between dermatologists and cosmetologists. Knowledge and shared experience will certainly bring the expected positive results of therapy [17]. For those reasons, the aim of this study was to investigate the efficacy of hydrogen purification in the treatment of acne vulgaris alone and in combination with cosmetic acids.

## 2. Materials and Methods

### 2.1. Study Design

A single-blind placebo study with follow-up analysis was conducted at the Institute of Health Sciences of the University of Opole, Poland, from January to March 2021 and at Medical College Yordanka Filaretova, Medical University of Sofia, Sofia, Bulgaria, from June to July 2021. The research was approved by the Human Research Ethics Committee of the Opole Medical School (No. KB/57/NOZ/2019) and conducted according to the principles of the Declaration of Helsinki. The study was registered at https://www.isrctn.com (No. ISRCTN 28257448) and accessed on 7 May 2020. The patients signed informed consent forms and agreed to take photos before and after the series of treatments. The participants knew that they could withdraw from the examination at any time. They did not have to give any reason of withdrawing.

**Figure 1:**
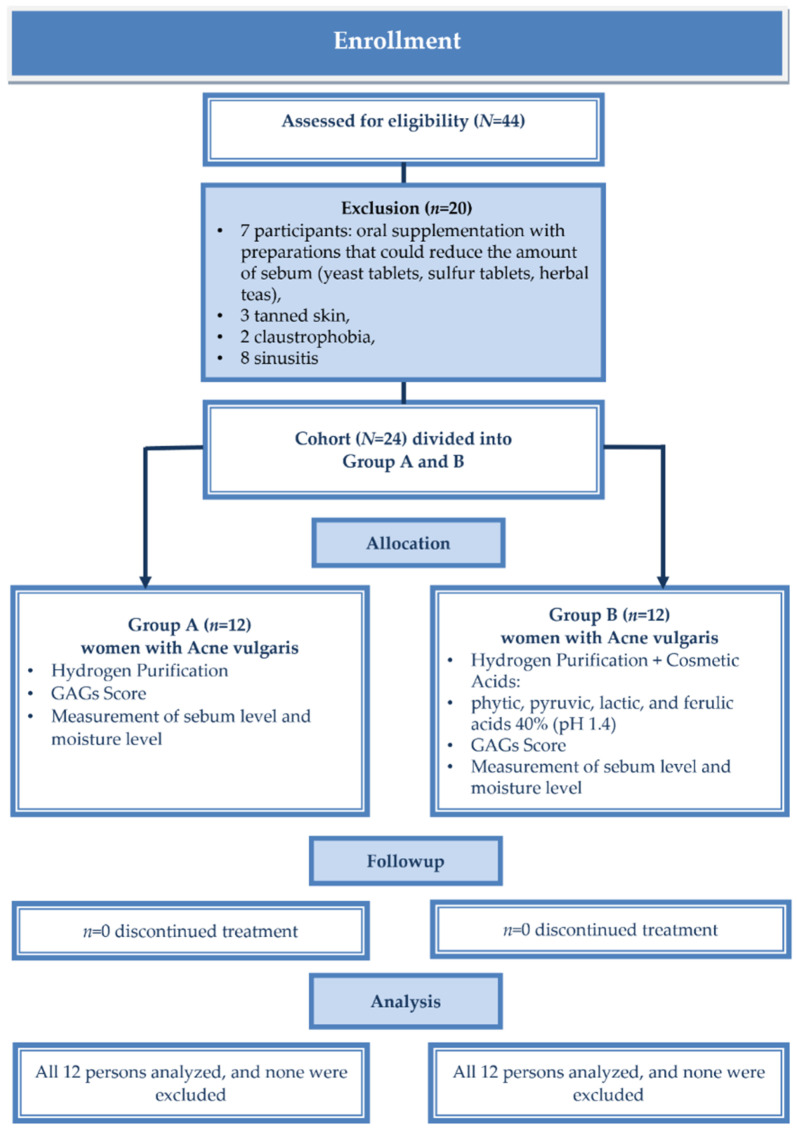
Patients follow in the study.

The G*Power ver. 3.1.9.6 software was used to determine a priori the appropriate sample size for the study. Considering 80% power and α = 0.05 (two-tail) for the repeated measures ANOVA with the interaction of within- and between-factors (2 groups, 2 time-points) the total sample size should equal *N* = 22. Initially, 44 people participated in the study, but 20 met the exclusion criteria (Figure 1). The final study sample consisted of 24 women. The power for the final sample size *N* = 24 was (1 − β) = 0.87 for the ANOVA.

No men participated in the study. Twenty-four women suffering from acne vulgaris were divided into two groups. They were assigned to the groups of a random selection of envelopes containing groups A or B. Group A underwent hydrogen purification using the H2Jet manipulator, which ejects alkaline water prepared by the device’s generator under pressure. Group B underwent hydrogen purification using the H2Jet manipulator with the subsequent use of a mixture of cosmetic acids (of phytic, pyruvic, lactic, and ferulic acids 40% (pH 1.4)).

All patients underwent a series of four treatments applied every 14 days. They could not have other cosmetic procedures. New cosmetics or sebum-regulating creams were forbidden during the entirety of the study period. Only cosmetics such as Cetaphil MD Dermoprotector, micellar water, SPF 50 cream, and Alantan Plus cream were approved to use.

### 2.2. Participants

A group of 24 women aged 19–21 (*M* = 20.13, *SD* = 0.80) who suffered from acne vulgaris were enrolled in the study. All participants were diagnosed with mild acne by the same rater dermatologist using the GAGS before and after a series of treatments.

The mean duration of acne was 6 years (*M* = 5.67, *SD* = 0.76), ranging between 5 and 7 years. Inclusion criteria for this study were: mild acne, no hormonal contraception, age 19–23 years, and no dermatological treatment within 12 months. The study had contraindications that made it impossible for some people to participate. For Group A it was: pregnancy, breastfeeding, claustrophobia, epilepsy, taking oral medications within the last 3 months, taking isotretinoin within the last year, active inflammation of the skin, fungal, viral, bacterial, allergic skin diseases, taking contraceptives, tanned skin, sun exposure after the procedure, psoriasis, eczema, skin cancers, numerous melanocytic nevi, atopic dermatitis, general illness, and a tendency to having sinusitis, numerous telangiectasias, recently having surgery (up to 2 months). For Group B it was: pregnancy, breastfeeding, claustrophobia, epilepsy, active inflammation of the skin, bacterial, viral, allergic, and fungal relapsing skin diseases, skin cancers, eczema, psoriasis, taking oral medications within the last 3 months, taking isotretinoin within the last year, taking contraceptives, sun exposure after the procedure, tanned skin, atopic dermatitis, general illness, and a tendency to having sinusitis, numerous telangiectasias, recently having surgery (up to 2 months), numerous melanocytic nevi, active herpes, reduced immunity, allergy to peeling ingredients, active rosacea, tanned skin, autoimmune diseases (pemphigus, collagenosis), having cryotherapy (up to 6 months), severe acne and propensity to keloids. Oral supplementation with yeast tablets, sulfur tablets, and herbal was forbidden.

Group A (*n* = 12) and B (*n* = 12) included young adult women with a higher sebum level (more than 100 μg/cm^2^) and acne vulgaris. Acne, excessive seborrhea, blackheads, whiteheads, and papules were observed in the volunteers. Before and after the treatment series, the GAGS was used to determine the severity of acne and to check whether the treatments had a positive effect on the improvement of the skin condition of the patients.

### 2.3. Measures

#### 2.3.1. Acne Vulgaris

The severity of acne vulgaris was assessed using the GAGS. Acne, excessive seborrhea, blackheads, whiteheads, and papules were observed. The GAGS scale is used to determine the degree of acne and to also check whether the treatments had a positive effect on the improvement of the skin [18].

#### 2.3.2. Skin Parameters

Before starting the tests and one month after the end of the last treatment, the skin parameters were measured. The participants were asked to remove face makeup the day before the measurements in the evening and not to apply any preparations to the face skin. In the morning the patients could not use any micellar fluid or cosmetics due to the fact that the parameters would be unreliable. Measurements were taken in the morning, with the test participants, after arriving in the room, acclimatizing for about 30 min. Room humidity was 40–50%, and the temperature was 20–21 degrees C. The Derma Unit SCC 3 apparatus (Courge & Khazaka, Cologne, Germany) was used for the test. Skin hydration was tested with the Corneometer CM 825, and sebum was measured using the Sebumeter SM 815. The measurement points were as follows: forehead, nose, right and left cheeks, and chin. The reliability of measurement was Cronbach’s α = 0.72 for moisturizing and Cronbach’s α = 0.73 for greasing in the study sample.

### 2.4. Treatment Procedure

Face makeup was removed in both groups (micellar fluid) before starting the treatments and also the skin was toned. Group A underwent a hydrogen purification treatment using specialized equipment generating water with an alkaline pH. After putting the subject on a cosmetic bed, the hair was covered with a cosmetic cap, cotton pads, and rubber goggles were put on the eyes, and the ears were covered and secured with cotton wool. Then, when the device was turned on, the vacuum was set to 2 bar, and alkaline water was ejected under pressure from the H2jet manipulator. The manipulator was held approximately 2–3 cm away from the subject’s skin, making epidermis exfoliation more precise. The exfoliation was carried out for about 5 min. The face was then dried with cosmetic wipes, and a sunscreen cream was applied to the treated skin. Treatment in group A was repeated in a series of 4 treatments every 2 weeks. Group B also underwent a hydrogen purification treatment, and the treatment algorithm was the same as that for group A. However, after drying the skin with a tissue, the skin was prepared with pre-peel cleanser and pre-peel lotion. Delicate areas such as the corners of the eyes, the area around the lobes of the nose, and the red lip were protected with petroleum jelly. Moistened cotton swabs were put on the eyes to protect against the penetration of acids into the eyeball. The acid was then applied to the entire treatment area of the face using a cotton baguette and left for 2 min during the first treatment. Each subsequent treatment had a longer exposure time to acid by 25 s. After treatment with acid, the neutralizer was applied to the skin for 1 min and then washed with cotton pads and cold water several times. Finally, the skin was dried, and a 50+ UV filter was applied to the face.

For home care, it was recommended to wash the face using Cetaphil MD Dermoprotector. After using the above-mentioned preparation, also, micellar fluid was allowed during the skin care. The patients were asked to use the Cetaphil MD Dermoprotector and micellar fluid twice a day for all periods of the study: in the morning and in the evening.

The patients were meant to apply Alantan Plus cream. Each patient used a thin layer of the cream in the morning and evening hours, after removing makeup.

The patients were instructed not to use the swimming pool and sauna and to avoid exposure to natural and artificial radiation. They were meant to use photoprotection every day and not use any preparations other than Cetaphil MD Dermoprotector, micellar fluid, and Alantan Plus cream. During the entire series of treatments, and a month after its completion, it was forbidden to use any other cosmetic and aesthetic medicine treatments. Dermatological procedures for the duration of the study were also prohibited.

### 2.5. Statistical Analysis

Initially, descriptive statistics (mean, standard deviation, median, skewness, kurtosis, and Shapiro–Wilk normality test) were performed to check the parametric properties of demographic variables, such as age and years of acne disease, and all dependent variables, such as the severity of acne (GAGS), face moisturizing and greasing, in both conditions before and after hydrogen purification treatment. Since the demographic variables did not meet the assumption of normal distribution of the data, the no-parametric Mann–Whitney *U*-test was conducted to check whether groups A and B of participants differed from each other. The effect of hydrogen purification treatment on the severity of acne and two parameters of face skin: moisturizing and greasing were examined using the repeated measures of one-way ANOVA. The Bonferroni post-hoc tests were performed to check the simultaneous statistically significant differences between the groups (A and B) and the conditions (before and after treatment). The effect size was assessed using η^2^*_p_* (with a 0.01 value interpreted as small, 0.06 as a medium, and above 0.14 as a large effect size). All statistics were performed using JASP software for Windows: JASP Team, Version 0.14.1 (Computer software; Amsterdam, The Netherlands: Department of Psychological Methods, University of Amsterdam; 2020).

## 3. Results

### 3.1. Changes in Acne Severity after Hydrogen Purification and Cosmetic Acids

Initially, the A and B groups were compared to each other to examine differences in age and acne years. Because age and acne years did not meet normality criteria, the Mann–Whitney *U*-test was performed for this purpose. Group A (Range 19–21, *M* = 20.00, *SD* = 0.85, *Mdn* = 20) did not differ significantly from group B (Range 19–21, *M* = 20.25, *SD* = 0.75, *Mdn* = 20) in age, *U* = 60, *Z* = −0.66, *p* = 0.51. No significant differences in years of acne were found between group A (Range 5–7, *M* = 5.75, *SD* = 0.75, *Mdn* = 6) and group B (Range 5–7, *M* = 5.58, *SD* = 0.79, *Mdn* = 5), *U* = 62, *Z* = 0.55, *p* = 0.58. So, both groups A and B did not differ in age and years of acne.

**Figure 2:**
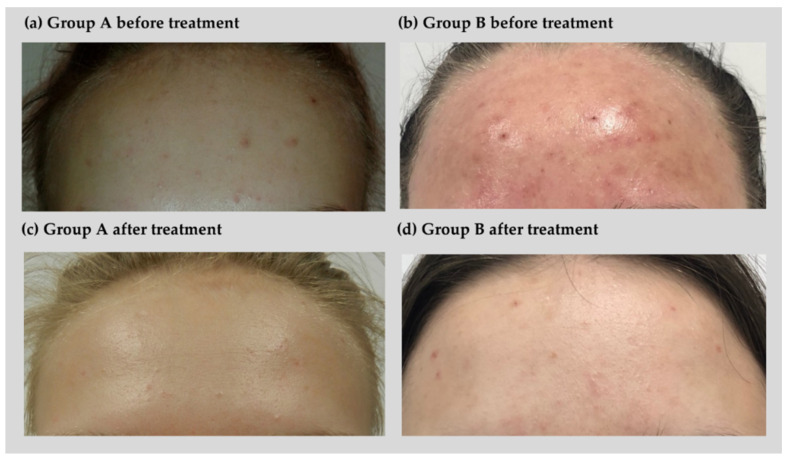
Effect of treatment on acne severity in a patient from group A before (**a**) and after treatment (**c**), group B before (**b**) and after treatment (**d**).

Acne severity before and after treatment was measured in groups A and B, before and after treatment, respectively (Table 1). The repeated measures one-way ANOVA showed that groups A and B differed significantly (*p* < 0.01) in acne severity with a large effect size. Statistically significant differences in acne severity were also found between conditions before and after hydrogen purification treatment (*p* < 0.001), with a large effect size. The interaction effect between the group and treatment was significant (*p* < 0.001) with a large effect size. A Bonferroni post-hoc test showed that Group A and B did not differ from each other in acne severity before treatment (*p* > 0.05). However, all groups differed significantly (*p* < 0.001) between conditions before and after treatment (with higher severity before than after treatment) and between groups A and B after treatment (with lower acne severity in group B than A), which is presented in Figure 2.

### 3.2. Changes in Facial Skin Moisture after Hydrogen Purification and Cosmetic Acids

The repeated measures one-way ANOVA was performed to examine the effect of hydrogen purification treatment on facial skin moisture in samples A and B. The results are presented in Table 2, separately for the forehead, nose, right and left cheek, and chin. The ANOVA showed no group, treatment, or interaction effect on forehead moisturizing in the women sample (*p* < 0.05).

The group effect was significant for nose moisturizing (*p* < 0.05), with a large effect size, indicating higher moisturizing in group B than A (Table 2). The treatment effect was significant (*p* < 0.001), with a large effect size, showing that the level of nose moisturizing was higher after treatment than before. In addition, the interaction effect between group and treatment was significant (*p* < 0.05), with a large effect size. The Bonferroni post-hoc test demonstrated that group B before treatment did not differ significantly from group A both before and after treatment (*p* > 0.05). The other differences between groups A and B before and after treatments were significant (*p* < 0.05), showing better nose moisturizing after treatment than before, with a greater effect in group B than A.

Regarding right check moisturizing (Table 2), ANOVA showed the significant effect of group (*p* < 0.01, large effect size) and treatment (*p* < 0.001, large effect size), but no interaction effect (*p* > 0.05, small effect size). Group A before treatment showed significantly lower right cheek moisturizing than groups A (*p* < 0.05) and B (*p* < 0.001) after treatment, as the Bonferroni post-hoc tests showed. The other differences between groups A and B before and after treatment were insignificant.

When the left check was examined using ANOVA (Table 2), all effects were significant, including group (*p* < 0.01, large effect size), treatment (*p* < 0.001, large effect size) and interaction (*p* < 0.01, large effect size) effects. Although group A did not differ from group B in left check moisturizing before the experiment (*p* > 0.05), the other differences between groups A and B before and after treatment were statistically significant (*p* < 0.001), showing a higher left check moisturizing after treatment than before, in particular among women from group B.

The ANOVA found significant group (*p* < 0.001, large effect size), treatment (*p* < 0.001, large effect size), and interaction (*p* < 0.01, large effect size) effects for check moisturizing among participants. The post-hoc test indicated that group B before treatment did not differ from group A before and after treatment, while the other differences between groups A and B before and after treatment were significant (*p* < 0.001), indicating higher check moisturizing after treatment, especially in the B group.

### 3.3. Changes in Facial Skin Greasing after Hydrogen Purification and Cosmetic Acids

The effect of hydrogen purification treatment on facial skin greasing was tested in samples A and B using repeated measures of one-way ANOVA (Table 3). The effects of group (*p* < 0.05, large effect size), treatment (*p* < 0.001, large effect size), and interaction (*p* < 0.01, large effect size) were significant for the forehead greasing. The post-hoc Bonferroni test showed that although groups A and B did not differ in forehead greasing before treatment (*p* > 0.05), all other differences between groups A and B before and after treatment were significant (*p* < 0.001), indicating lower forehead greasing after treatment than before, especially in the B group (Table 3).

Next, the nose greasing was examined before and after treatment in groups A and B, using ANOVA (Table 3). All effects were significant, including group (*p* < 0.01, large effect size), treatment (*p* < 0.001, large effect size), and interaction (*p* < 0.001, large effect size) effects. The Bonferroni post-hoc tests indicate that groups A and B before treatment did not differ from each other significantly (*p* > 0.05), but the other differences between samples A and B before and after treatment were significant (*p* < 0.001). The levels of nose greasing were significantly lower after treatment than before in both samples, but this effect was statistically stronger in group B compared to group A.

The ANOVA did not show any significant effect of group (A vs. B), treatment (before vs. after), and interaction between group and treatment on the greasing of the right cheek (*p* > 0.05). In contrast, significant effects of group (*p* < 0.01, large effect size), treatment (*p* < 0.001, large effect size), and interaction (*p* < 0.05, large effect size) were found on the left cheek greasing. Although no differences were demonstrated between groups A and B before treatment, the other differences between samples A and B, considering conditions before and after treatment, were significant (*p* < 0.001). More specifically, the level of left cheek greasing decreased after treatment, particularly among women in group B (see Table 3 for more details).

The significant effects of group (*p* < 0.01, large effect size) and treatment (*p* < 0.001, large effect size), but no interaction between group and treatment (*p* > 0.05), were shown for chin greasing in the A and B samples (Table 3). Apart from no differences between samples A and B before treatment, all other significant differences were found between groups (A and B) and conditions (before and after treatments) in the study (*p* < 0.001). Compared to the condition before the experiment, lower chin greasing was presented after treatment in both A and B samples, with a greater difference in group B than A.

## 4. Discussion

The results of the research presented in this article show that cosmetological treatments have a positive effect on improving basic skin parameters and reducing skin eruptions that occur in people struggling with acne. Furthermore, the synergy of two treatments is definitely a better solution and gives better results than the use of single cosmetic methods. The research conducted first by Chilicka et al. in 2021 on the use of hydrogen purification in people suffering from acne showed that the procedure is safe and improves the skin condition of the study participants [11]. To the best of our knowledge, there are no other reports in the literature on the influence of alkaline water on the skin of people with acne.

Much more is known about the use of cosmetic acids, as treatments with their use have been known in dermatology and cosmetology for many years. Cosmetic acids provide very good results in terms of reducing skin eruptions and restoring proper skin parameters. Sarkar et al. conducted a series of treatments involving 45 patients with acne. Group A underwent biweekly peeling sessions with 35% glycolic acid, group B underwent treatment with 20% salicylic–10% mandelic acid treatment, and group C received phytic acid peels for a total of six sessions. After 12 weeks of treatment, there was a significant reduction in the number of inflammatory and non-inflammatory eruptions in all groups. The reduction in the acne scores in the three study groups were 70.55%, 74.14%, and 69.7%, respectively [19].

Chilicka et al. conducted research on a group of 120 young women. Group A (*n* = 60) underwent Azelaic Peeling treatment (6 sessions, every 2 weeks), and group B (*n* = 60) underwent Pyruvic Peeling treatment (6 sessions, every 2 weeks). Both Azelaic Peeling and Pyruvic Peeling produced a significantly lower desquamation level. However, significant differences between these two agents were shown in the extent of the oily skin level [20].

Kamm et al. compared the effect of ferulic acid and the use of d’Arsonwal’s high-frequency currents on acne-prone skin. Group A (*n* = 30) had a series of 5 treatments with ferulic acid performed every 7 days. The acid was applied to the face for 6 min. Group B (*n* = 30) had the same series of treatments but with the use of d’Arsonwal’s high-frequency currents. With probants in the ferulic acid group, the number of inflammatory exanthema after the end of the treatment was significantly lowered. In d’Arsonwal’s current group, the number of inflammatory exanthema also significantly reduced [21].

An interesting study was conducted by Marczyk et al. who compared the effect of the application of 50% pyruvic and 30% salicylic peels on the skin in patients with acne vulgaris. Ten patients were treated with 50% pyruvic acid and 10 patients with 30% salicylic acid. The series of the treatment included five treatments performed every two weeks. To measure the amount of sebum on the surface of the epidermis the Sebumeter SM 815 device was used. Better results in terms of sebum reduction were obtained in the group in which salicylic peel was used [22].

Jaffary et al. conducted a study with the use of the same acids at the same concentrations. The study group consisted of 86 patients randomly divided into two groups. Routine acne treatment (4% topical solution of erythromycin, triclocarban soap, and sunscreen) was used twice a day for 8 weeks. Additionally, 30% salicylic acid was used for the control group and 50% pyruvic acid for the study group. The reduction in the number of comedones, papules, and acne severity index was statistically significant (*p*< 0.001) in the course of treatment in both groups [23].

In another study by Zdrada et al. 50% pyruvic acid and preparation containing glycolic and salicylic acids were used. Fourteen women with a diagnosis of acne underwent a series of four treatments at 2-week intervals. Pyruvic acid was applied to the right side of the patient’s face, and glycolic and salicylic acids were applied to the left side. Basic skin parameters such as hydration, sebum secretion, and skin color were evaluated. The increase in skin hydration on the left side of the chin and nose was not statistically significant. Treatment with a mixture of acids resulted in fewer side effects than a single acid used in high concentrations, but therapeutic effects were comparable [24].

In cosmetology, many treatments for acne-prone skin benefit from using modern devices, including those that are based on cosmetic acids. One of them is microdermabrasion. It is safe and effective without excluding the patient from his/her everyday life. The above-mentioned treatments have a positive effect on the reduction of acne lesions, reduction of the level of sebum on the surface of the epidermis, and improvement of skin hydration [25,26]. We hope that our research results and the treatments that we have proposed, can be used by other researchers to apply them to another disease, for example hidradenitis suppurativa [27,28,29].

## 5. Study Limitations

In the future, larger study samples should be carried out that include both genders and a wider age range. We would like to include additional measurements, such as pH. We could also compare the effectiveness of other cosmetic treatments or other types of cosmetic acids.

## 6. Conclusions

Hydrogen purification is a procedure that is increasingly used in cosmetology. Our research shows that it produces very good results in terms of reducing skin eruptions and improving skin parameters. The synergy of the treatments, namely hydrogen purification and cosmetic acids, indicates that they provide better results than their use as individual treatments. These procedures do not require patients to abandon their daily activities. Finally, they do not cause side effects such as redness or skin irritation. However, it should be remembered that dermatological treatment cannot be replaced by cosmetological treatment.

## Figures and Tables

**Table 1:** Repeated measures of one-way ANOVA for acne severity in groups A and B, before and after treatment, using the Global Acne Grading System (GAGS).

GAGS	Group A		Group B		Effect	*F*(1, 22)	*p*	η^2^*_p_*
	*M*	*SD*	*M*	*SD*				
					G	12.32	0.002	0.359
Before treatment	16.42	1.00	16.50	1.00	T	430.26	<0.001	0.951
After treatment	11.58	1.51	9.00	0.95	T × G	20.11	<0.001	0.478

Note. Group A = hydrogen purification, Group B = hydrogen purification and cosmetic acid, GAGS = Global Acne Grading System, *M* = mean, *SD* = standard deviation, *F* = Fisher’s test, *p* = significance level, η^2^*_p_* = partial eta-square effect size, G = group effect, T = treatment effect, T × G = interaction between group and treatment effect.

**Table 2:** Repeated measures of one-way ANOVA for face moisturizing in groups A and B, before and after treatment, using Corneometer CM825 (g/m^2^).

Face Moisturizing	Group A		Group B		Effect	*F*(1, 22)	*p*	η^2^*_p_*
	*M*	*SD*	*M*	*SD*				
**Forehead**					G	2.01	0.170	0.08
Before	39.83	3.53	41.09	3.20	T	0.00	0.991	0.00
After	39.91	2.32	41.03	1.63	T × G	0.01	0.922	0.00
**Nose**					G	5.30	0.031	0.19
Before	39.91	2.32	41.03	1.63	T	39.89	<0.001	0.65
After	42.08	3.53	45.53	3.14	T × G	4.91	0.037	0.18
**Right Cheek**					G	8.72	0.007	0.28
Before	38.78	3.49	42.96	2.87	T	15.20	<0.001	0.41
After	44.50	4.00	46.48	4.82	T × G	0.86	0.363	0.04
**Left Cheek**					G	13.63	0.001	0.38
Before	40.85	3.21	41.64	2.40	T	94.92	<0.001	0.81
After	45.73	3.66	51.53	1.89	T × G	10.96	0.003	0.33
**Chin**					G	14.97	<0.001	0.41
Before	38.90	3.41	40.93	2.18	T	111.46	<0.001	0.84
After	43.64	3.27	49.58	2.83	T × G	9.52	0.005	0.30

Note. Group A = hydrogen purification, Group B = hydrogen purification and cosmetic acid, *M* = mean, *SD* = standard deviation, *F* = Fisher’s test, *p* = significance level, η^2^*_p_* = partial eta-square effect size, G = group effect, T = treatment effect, T × G = interaction between group and treatment effect.

**Table 3:** Repeated measures one-way ANOVA for face greasing in groups A and B, before and after treatment, using Sebumeter SM815 (μg/cm^2^).

Face Greasing	Group A		Group B		Effect	*F*(1, 22)	*p*	η²*_p_*
	*M*	*SD*	*M*	*SD*				
**Forehead**					G	7.68	0.011	0.26
Before	191.58	17.55	189.58	14.58	T	454.62	<0.001	0.95
After	127.25	15.16	103.08	9.23	T × G	9.82	0.005	0.31
**Nose**					G	7.96	0.010	0.27
Before	185.33	19.42	189.08	17.43	T	366.86	<0.001	0.94
After	130.67	8.13	100.50	10.72	T × G	20.57	<0.001	0.48
**Right Cheek**					G	0.49	0.490	0.02
Before	191.42	20.22	191.33	16.82	T	0.49	0.491	0.02
After	194.33	19.34	184.25	20.10	T × G	2.82	0.107	0.11
**Left Cheek**					G	8.23	0.009	0.27
Before	194.33	19.34	184.25	20.10	T	351.12	<0.001	0.94
After	134.17	16.79	108.92	12.81	T × G	4.40	0.048	0.17
**Chin**					G	8.32	0.009	0.27
Before	191.83	15.95	186.17	17.79	T	177.16	<0.001	0.89
After	135.25	13.84	117.75	12.40	T × G	1.59	0.221	0.07

Note. Group A = hydrogen purification, Group B = hydrogen purification and cosmetic acid, *M* = mean, *SD* = standard deviation, *F* = Fisher’s test, *p* = significance level, η^2^*_p_* = partial eta-square effect size, G = group effect, T = treatment effect, T × G = interaction between group and treatment effect.

## Data Availability

Not applicable.
